# Comparison of Polydioxanone and Polypropylene Sutures in Columellar Incision Closure: A Prospective Randomized Clinical Trial

**DOI:** 10.1007/s00266-026-05852-w

**Published:** 2026-04-13

**Authors:** Tankut Uzun, Eray Uzunoğlu, Mehmet Ekrem Zorlu, Berrak Karatan, Togay Müderris

**Affiliations:** 1 Department of Otolaryngology and Head & Neck Surgery, Cigli Training and Research Hospital, Bakircay University Faculty of Medicine, Izmir, Turkey; 2 Department of Otolaryngology and Head & Neck Surgery, Gazi University Faculty of Medicine, Ankara, Turkey; 3 Department of Plastic Reconstructive and Aesthetic Surgery, Istanbul University Cerrahpasa Faculty of Medicine, Istanbul, Turkey

**Keywords:** Open rhinoplasty, Columella, Scar, Polydioxanone, Polypropylene, Suture

## Abstract

**Background:**

Columellar scarring remains an important aesthetic concern in open septorhinoplasty, and while multiple studies have compared absorbable and non-absorbable sutures, the specific effects of slowly absorbable polydioxanone (PDS) on long-term scar outcomes have not been previously investigated.

**Materials and Methods:**

Ninety-six patients undergoing primary septorhinoplasty between January 2023 and January 2024 were randomly assigned to the PDS (*n* = 47) or Prolene (*n* = 49) groups. Patients with prior nasal surgery or systemic connective tissue disorders were excluded. Scar appearance was evaluated one year postoperatively using the Stony Brook Scar Evaluation Scale (SBSES) and a 0–100 visual analog scale (VAS) rated by both patients and a blinded otolaryngologist.

**Results:**

There were no statistically significant differences in patient VAS scores (*P* = 0.326), physician VAS scores (*P* = 0.055), or SBSES scores (*P* = 0.41) between the groups. Confidence-interval–based non-inferiority analyses supported non-inferiority for physician-rated VAS, whereas patient-rated VAS and SB total were close to the prespecified margins.

**Conclusion:**

PDS sutures yield comparable aesthetic outcomes to Prolene and offer the additional benefits of absorbability and sustained tensile strength. They may be preferable in cases where long-term support is needed or where suture removal may cause discomfort.

**Level of Evidence I:**

This journal requires that authors assign a level of evidence to each article. For a full description of these Evidence-Based Medicine ratings, please refer to the Table of Contents or the online Instructions to Authors www.springer.com/00266.

## Introduction

Open rhinoplasty is a commonly preferred approach among surgeons due to its superior surgical exposure and the ease with which the necessary maneuvers can be performed. Despite these advantages, postoperative scarring at the columellar incision site may negatively affect patient satisfaction and can be aesthetically bothersome [1]. Although various incision techniques have been developed, Goodman’s classic inverted V incision remains widely favored, as it is believed to minimize visible scarring. In this approach, efforts are generally directed toward reducing scar formation by placing the incision at the narrowest point of the columella and employing appropriate suture materials and techniques [1, 2]. Non-absorbable sutures such as polypropylene are commonly preferred for the closure of columellar incisions due to their low inflammatory response and ability to maintain optimal wound tension. However, their use is associated with notable drawbacks, including the requirement for suture removal, patient discomfort during the procedure [3].

In contrast, rapidly absorbable sutures such as polyglycone and polyglactin are increasingly favored for their advantages in patient comfort, as they eliminate the need for removal and save office time [3, 4]. Polydioxanone (PDS) is a slowly absorbable suture material that provides prolonged wound support and superior tensile strength compared to other rapidly absorbable alternatives [5, 6].

Although numerous studies in the literature have compared the effects of absorbable and non-absorbable suture materials on similar columellar scar outcomes, it is generally accepted that both types can be used safely and effectively [3, 4, 7]. However, to our knowledge, there are no studies specifically evaluating the impact of PDS sutures on columellar scarring.

In this study, we aimed to evaluate PDS as a potential absorbable suture alternative in all primary rhinoplasty cases by comparing its effects on long-term columellar scar formation with those of polypropylene, a non-absorbable material that has long been considered reliable. Another objective of the data to be presented is to contribute to the literature by supporting the development of an absorbable suture material that can be safely used in selected cases requiring substantial projection enhancement and prolonged skin tension.

## Materials and Methods

This study was designed as a prospective randomized clinical trial including patients aged 18 to 50 years who presented to the Otorhinolaryngology Clinic of Bakircay University between January 2023 and January 2024 for primary septorhinoplasty. Ethical approval was obtained from the Bakircay University Ethics Committee. The study was conducted in accordance with the principles of the Declaration of Helsinki, and written informed consent was obtained from all participants.

Patients with a history of previous nasal surgery for aesthetic or oncological purposes, as well as those with systemic connective tissue disorders, were excluded from the study. Participants were randomized to undergo columellar incision closure with either absorbable or non-absorbable sutures using a computer-generated randomization protocol.

The primary analysis was framed as a non-inferiority comparison of PDS versus polypropylene for columellar scar outcomes. Assuming a one-sided α = 0.025 and 90% power, the required per-group sample size for the SBSES total score was 45 when using a clinically acceptable non-inferiority margin of 0.50 points and a pooled SD of 0.57 derived from prior data. For the VAS outcome, using a pooled SD of 8.8 and a non-inferiority margin of 6 points on the 0–100 VAS Scale (reflecting a difference below the threshold of clinical relevance), the required per-group sample size was 46.

A total of 100 patients were initially enrolled. Four patients who were lost to follow-up were excluded, and the study was completed with 96 patients: 47 in the PDS 6-0 suture (PDS II, Ethicon Inc., Somerville, NJ, USA) group and 49 in the polypropylene (Prolene 6/0; Ethicon Inc., Somerville, NJ, USA) group.

### Surgical Technique

**Fig. 1:**
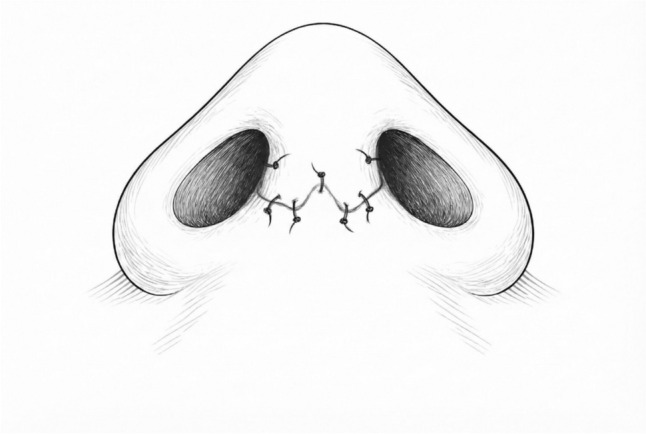
Inverted V incisions of the columella and skin sutures

All patients were operated under general anesthesia by the same surgeon. Open technique septorhinoplasty was performed following standard surgical steps and employing Goodman’s classic inverted V-shaped incision. Closure of the columellar incision was achieved using seven externally placed interrupted sutures with either PDS in Group 1 or polypropylene in Group 2 (Fig. 1). All closures were performed using the same externally placed interrupted suture technique, with evenly spaced sutures and careful tension control. Excessive knot tightening and wound-edge constriction were avoided, and no complications attributable to either suture material were observed. No additional suture techniques or materials were employed. Polypropylene sutures were removed on postoperative day 7. In the PDS group, only suture tails long enough to cause discomfort were trimmed, and the knots were left in situ. All patients received identical postoperative management and follow-up. Patients were instructed to apply Terramycin® (Pfizer) antibiotic cream twice daily for the first 7 days. Thereafter, only Bepanthol Derma® (Bayer) was applied three times daily until the 1-month follow-up. At the 1-month follow-up, all knots in the clear PDS monofilament sutures had become untied. Any visible, loosened ends were gently removed by simple traction to eliminate protruding threads; no cutting or formal suture removal was required. This step was performed solely to remove visible remnants and optimize patient satisfaction. No additional wound-care measures were prescribed. It was confirmed that no patient received additional care beyond the study protocol. Routine follow-ups were conducted at 1, 3, and 6 months, with the final assessment at 12 months.

### Data Collection

**Fig. 2:**
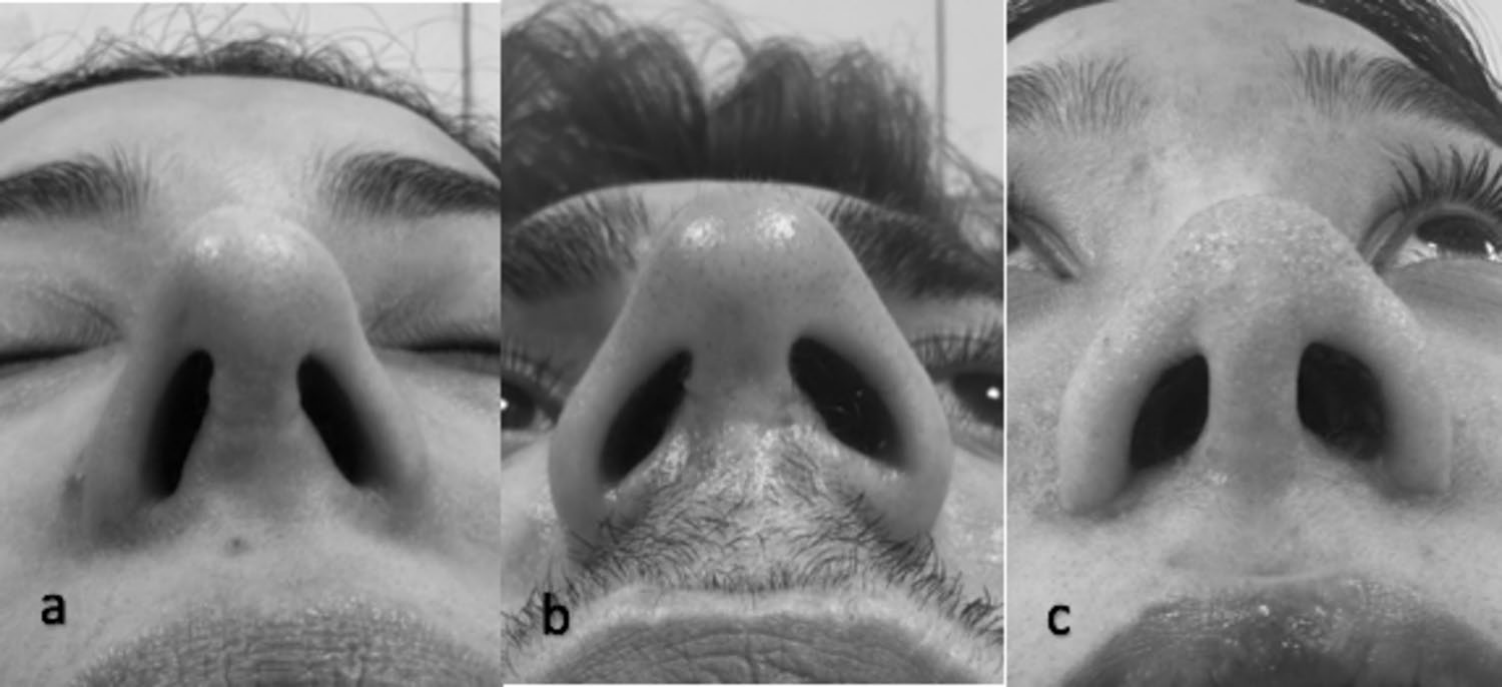
Examples of columellar scar outcomes: **a** height irregularity, either depressed or elevated relative to the surrounding skin; **b** notching; and **c** normal, satisfactory result

Postoperative basal view and profile photographs were taken and recorded at the one-year follow-up. Patients were evaluated by a blinded otolaryngologist using the Stony Brook Scar Evaluation Scale (SBSES), adapted for columellar scar assessment at the same time [8] (Fig. 2). In addition, patients were asked to rate the appearance of their scars using a visual analog scale (VAS) ranging from 0 to 100, with 100 indicating optimal wound healing. The same VAS was also independently completed by the same blinded otolaryngologist to assess scar outcomes.

### Statistical Analysis

Statistical analyses were performed using SPSS version 25.0 (IBM Corporation, Armonk, NY, USA). The normality of data distribution was assessed using the Kolmogorov-Smirnov and Shapiro-Wilk tests. Group comparisons for VAS and SBSES scores were conducted using the Mann-Whitney U test. Pearson’s chi-square test and Fisher’s exact test were used to compare categorical parameters (thickness, pigmentation, notching, leveling-presence or absence of these pathological features) between Group 1 and Group 2. Patient-reported VAS scores and otolaryngologist-assessed VAS scores were compared using the Wilcoxon signed-rank test. Quantitative variables are presented as mean ± standard deviation (SD) and median (min–max), while categorical variables are expressed as frequencies and percentages (*n*, %). A *P*-value of < 0.05 was considered statistically significant. Non-inferiority was assessed using the prespecified margins (0.50 points for SBSES and 6 points for VAS). Non-inferiority of PDS versus polypropylene was concluded if the lower bound of the two-sided 95% confidence interval for the between-group difference (PDS−polypropylene) was greater than − 0.50 for SBSES and greater than − 6 for VAS.

## Results

The demographic characteristics of the patients are presented in Table 1. The groups were similar in terms of age, sex and Fitzpatrick skin types [9] (2/3/4) (all *P* > 0.05). None of the participants experienced excessive bleeding, unexpected infection during wound healing, adverse tissue reaction or columella necrosis at any time during the follow-up period.

**Table 1:** Demographic characteristics by suture groups (PDS vs Prolene)

		PDS Group (*n* = 47)	Prolene Group (*n* = 49)	Total (*n* = 96)
Age (mean ± SD)		26.30 ± 5.54	27.53 ± 8.88	26.93 ± 7.43
Sex (n, %)	Female	36 (76.6%)	33 (67.3%)	69 (71.9%)
	Male	11 (23.4%)	16 (32.7%)	27 (28.1%)
Fitzpatrick Skin Type (n, %)	Type 2	5 (10.6%)	6 (12.2%)	11 (11.5%)
	Type 3	21 (44.7%)	22 (44.9%)	43 (44.8%)
	Type 4	21 (44.7%)	21 (42.9%)	42 (43.8%)

Values are shown as *n* (%) for categorical variables and mean ± SD for age

Consistently high VAS scores were observed in both patient- and physician-based assessments (Table 2). There was no statistically significant difference in the comparison between the patients' VAS scores and the doctor's VAS scores (*P* = 0.9).

**Table 2:** VAS scores

	PDS group	Prolene group	*P* value
Patient VAS Score	92,87±10,46	95,36±7,03	0.326
Doctor VAS Score	95,53±6,18	93,46±6,14	0.055

The SBSES evaluation results are presented in Table 3. Comparisons of both the mean patient scores and the number of patients with pathological findings according to the scale revealed no statistically significant differences between the groups.

**Table 3:** Stony Brook scar evaluation scale (SBSES) results

SBSES		PDS group		Prolene group		*P* value
		Score*	Patient(*n* = 47)*	Score*	Patient(*n* = 49)*	
Width	> 2 mm	1	0	1	0	*P* = 1
	< 2 mm		47		49	
Height	Depressed or elevated from surrounding skin	0.87±0.33	6	0.85±0.35	7	*P* = 0.82
	Flat		41		42	
Discoloration	Darker than surrounding skin	0.97±0.14	1	0.95±0.19	2	*P* = 0.58
	Same color or lighter		46		47	
Notching	Present	0.87±0.33	6	0.93±0.24	3	*P* = 0.26
	Absent		41		46	
Overall appearance	Poor	0.97±0.14	1	1	0	*P* = 0.3
	Good		46		47	
Total Scores		4.72±0.71		4.87±0.38		*P* = 0.41

^*^Columellar scars were assigned 0 or 1 point each for the presence or absence. Comparisons of SBSES scores between groups were performed using the Mann–Whitney U test. The number of patients with pathological findings was compared using the Pearson chi-square test, with confirmation of the relevant parameters conducted via the Fisher’s exact test. Identical *P*-values were obtained for both comparisons, as presented in the table

Between-group differences were small. Confidence-interval–based non-inferiority analyses supported non-inferiority for doctor-rated VAS; however, patient-rated VAS and SB total estimates were close to the prespecified margins, with lower bounds marginally crossing the non-inferiority thresholds (Tables 2 and 3). For doctor-rated VAS, the between-group difference (PDS−polypropylene) was Δ = 2.06 (95% CI − 0.42 to 4.48), supporting non-inferiority relative to the prespecified margin of − 6 points. For patient-rated VAS, the difference was Δ = − 2.50 (95% CI − 6.13 to 0.93), with the lower bound marginally crossing the prespecified non-inferiority margin. For SB total, the difference was Δ = − 0.24 (95% CI − 0.53 to 0.02), with the lower bound close to the − 0.50 margin.

## Discussion

Despite the numerous advantages of open septorhinoplasty for the surgeon, the columellar incision scar remains a concerning postoperative outcome. Minimizing such scarring requires the selection of optimal incision techniques and appropriate suture materials [1, 2]. To this end, numerous studies in the literature have examined the selection of appropriate suture materials to minimize columellar scarring [7, 10, 11].

A meta-analysis of six studies involving a total of 435 patients, which compared absorbable and non-absorbable suture materials, concluded that the type of suture material had no effect on long-term columellar scarring [7]. In a recent study involving 41 patients (23 with rapidly absorbable sutures and 18 with non-absorbable sutures), no association was found between the type of suture material and columellar scar formation [10]. Another recent study investigating the effects of rapidly absorbable and non-absorbable sutures on columellar scars found no difference in the evaluated short-term outcomes [11].

Similarly, Kılavuz et al. [12]. compared Rapid Vicryl and Prolene sutures with respect to columellar incision scars, using VAS and SBSES scores, and likewise reported no significant differences.

All of the aforementioned studies compared rapidly absorbable suture materials with non-absorbable materials. In our study, we evaluated PDS sutures, a more slowly absorbable material, in comparison with Prolene sutures, a non-absorbable material, in terms of columellar scar outcomes. Consistent with recent studies, we observed no differences in scar VAS scores assessed by either patients or physicians. Moreover, there were no statistically significant differences in SBSES scores or in the number of patients presenting with pathological findings (scar width, height, discoloration or notching).

To our knowledge, no previous study has examined the effect of slowly absorbable PDS sutures on columellar incision scars. Our findings therefore provide a noteworthy contribution to the literature in this regard. The absence of infection or tissue reaction in any of our patients provides important evidence supporting the safe use of this suture material.

Polyglactin 910, a rapidly absorbable suture material frequently compared with Prolene in the literature, is considered a safe option for the closure of columellar incisions. PDS, which dissolves more slowly than polyglactin, offers greater tissue strength and tension while demonstrating comparable tissue compatibility and resorption characteristics [13, 14].

In another recent study, PDS and polyglactin-910, a rapidly absorbable suture material, were compared as intradermal sutures in patients undergoing surgical scar revision, with superior scar outcomes observed in the PDS group [5].

In light of these findings, we believe that polydioxanone suture material, which we have demonstrated to be safe for use on columellar scars in primary rhinoplasty, can be particularly advantageous in cases where prolonged suture tension and strength are required.

### Limitations

This study has several limitations. It was conducted at a single center by a single operating surgeon, which may limit external validity. Although the total sample size (n = 96) met the a priori non-inferiority target for SBSES and approached the target for VAS, the study was underpowered to detect small between-group differences and did not allow meaningful subgroup analyses (e.g., by sex or Fitzpatrick skin type). The cohort was restricted to primary rhinoplasty without prior nasal surgery or connective-tissue disorders, limiting generalizability to revision procedures and higher-risk populations. Finally, health-economic and patient-experience outcomes (e.g., time, cost, and discomfort associated with suture removal) were not captured, precluding comparative cost–benefit inferences between absorbable and non-absorbable materials.

## Conclusion

Based on the findings of our study, PDS is an important absorbable suture material that can be safely used for the closure of columellar incision scars in primary rhinoplasty.
